# Mid-term outcomes of the COMMENCE trial investigating mitral valve replacement using a bioprosthesis with a novel tissue

**DOI:** 10.1016/j.xjon.2023.05.008

**Published:** 2023-06-02

**Authors:** David A. Heimansohn, Craig Baker, Evelio Rodriguez, Hiroo Takayama, Francois Dagenais, David S. Talton, Mubashir A. Mumtaz, Philippe Pibarot, John D. Puskas, COMMENCE Trial Investigators

**Affiliations:** a St Vincent The Heart Center of Indiana, Indianapolis, Ind; b Depertament of Cardiothoracic Surgery, Cardiovascular Thoracic Institute, University of Southern California, Los Angeles, Calif; c Ascension Saint Thomas Heart, Nashville, Tenn; d Columbia University Irving Medical Center, New York Presbyterian Hospital, Division of Cardiothoracic and Vascular Surgery, New York, NY; e North Mississippi Medical Center, Tupelo, Miss; f Institut Universitaire de Cardiology et Pneumologie de Quebec, Quebec, Canada; g Department of Cardiovascular and Thoracic Surgery, UPMC Central Pennsylvania, Harrisburg, Pa; h Department of Cardiology, Québec Heart and Lung Institute, Laval University, Quebec, Canada; i Department of Cardiovascular Surgery, Mount Sinai Morningside, New York, NY

**Keywords:** mitral valve replacement, mitral valve diseases, bioprosthetic valve, RESILIA tissue

## Abstract

**Objective:**

Novel tissue leaflets (RESILIA tissue) may improve durability of bioprosthetic heart valves. The COMMENCE trial is an ongoing prospective study to evaluate valve replacement using RESILIA tissue. This report describes mid-term outcomes in the mitral cohort of COMMENCE.

**Methods:**

Adult patients requiring mitral valve replacement were enrolled in a prospective, single-arm trial at 17 sites in the United States and Canada. An independent clinical events committee adjudicated safety events using definitions from established guidelines, and hemodynamic performance was evaluated by an independent echocardiographic core laboratory.

**Results:**

Eighty-two patients (median age 70 years) successfully underwent mitral valve replacement with the study valve. Five-year event-free probabilities for all-cause mortality, structural valve deterioration, and reoperation were 79.9%, 98.7%, and 97.1%, respectively. Hemodynamic valve function measurements were stable through the 5-year follow-up period; valvular leaks were infrequently observed and primarily clinically insignificant/mild.

**Conclusions:**

Mitral valve replacement patients implanted with a RESILIA tissue bioprosthesis had a good safety profile and clinically stable hemodynamic performance.

## Graphical abstract

**Figure:**
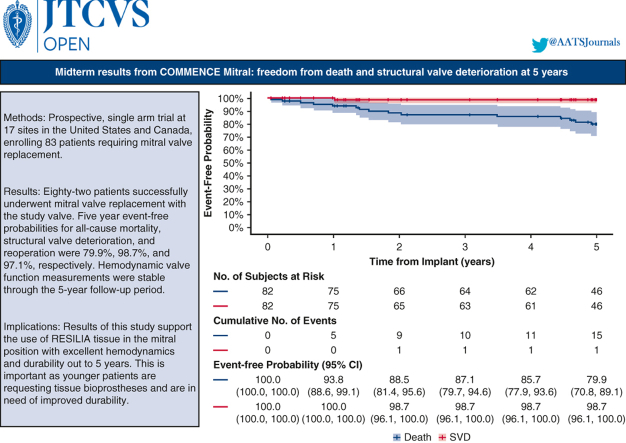

**Figure:**
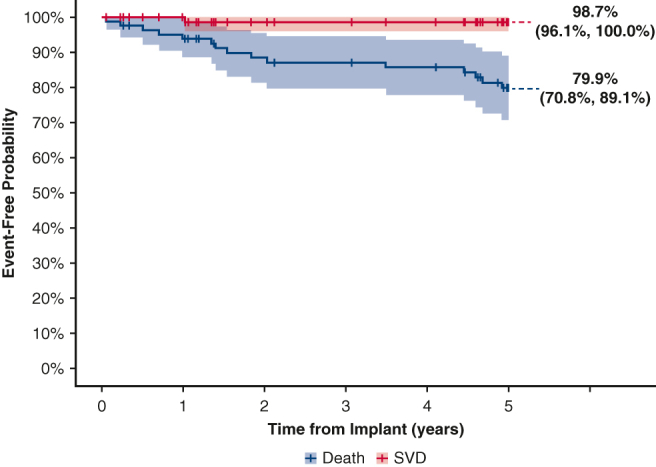
Freedom from death and structural valve deterioration at 5 yrs. in COMMENCE mitral patients.

**Central Message**

Through 5 years of patient follow-up, mitral valve replacement patients implanted with a RESILIA tissue bioprosthesis had a good safety profile and clinically stable hemodynamic performance.

**Perspective**

Patient preferences in mitral valve replacement are evolving, as the use of anticoagulation falls out of favor; meanwhile, reoperations have become safer with catheter-based valve-in-valve replacements a reality. Improved durability is the focus of new tissue valve designs. This study reports outcomes of a new pericardial tissue designed to reduce leaflet calcification and improve long-term durability.

Mitral valve disease is one of the most common valvular heart diseases, with functional classifications of mitral regurgitation together affecting an estimated 5% of the US population and resulting in approximately 29,000 hospitalizations in the United States in 2018.^1^ Further, prevalence is increasing in developed nations as the population ages.^2^ Current valvular heart disease guidelines establish that mitral valve replacement (MVR) may be considered in patients with mitral regurgitation and mitral stenosis when durable repair is not feasible.^3^ The optimal prosthetic valve in MVR remains a matter of debate, particularly for patients younger than 65 years of age.^3^, ^4^, ^5^ Patients can be implanted with either a bioprosthetic valve or a mechanical valve. Mechanical valves may offer longer durability but require lifelong anticoagulation with a potential risk of bleeding or pannus formation with leaflet restriction depending on the adequacy of anticoagulation.^6^ In contrast, bioprosthetic valves have been associated with greater risk of structural valve deterioration (SVD), especially in younger patients, and with reduced overall survival in patients younger than 50 years.^7^^,^^8^ The potential of valve-in-valve therapies may reduce the need for redo MVR, thereby impacting the likelihood of younger patients receiving a bioprosthetic valve. Taken together, balancing patient quality of life and the competing risks of reoperation and potential bleeding events requires long-term data to inform decision-making on appropriate valve selection.^9^

RESILIA tissue designed for use in heart valve replacement is a promising option that may improve durability of bioprosthetic valves. The tissue incorporates a novel integrity preservation technology, which prevents calcium binding through stable capping of residual aldehyde groups and allows dry tissue storage via glycerolization. RESILIA tissue has exhibited reduced tissue calcification in preclinical studies compared with both previous bovine tissue preparations and porcine tissues treated with amino oleic acid.^10^ When evaluated in a juvenile sheep model, RESILIA tissue valves implanted in the mitral position exhibited reduced transvalvular pressure gradients and approximately 72% less calcium relative to the PERIMOUNT control group after 8 months.^11^

Clinically, the European Aortic Feasibility Study investigated RESILIA tissue in aortic valve replacement with a good hemodynamics profile and no SVD observed through 5 years of follow up.^12^ The COMMENCE Investigational Device Exemption trial was planned to evaluate performance of RESILIA tissue in both aortic and mitral valve replacement. Promising outcomes have been observed through 5 years of follow-up in the aortic position with clinically stable hemodynamics and no evidence of SVD reported.^13^ Herein, we report mid-term outcomes of the mitral cohort of the COMMENCE trial. The objective of this study was to evaluate safety and effectiveness of mitral valve replacement with RESILIA tissue.

## Methods

The COMMENCE trial was designed as a prospective, single-arm Food and Drug Administration Investigational Device Exemption trial with enrollment of the mitral cohort at 17 sites in the United States and Canada. Patients underwent MVR with Model 11000M (Edwards Lifesciences LLC), a pericardial mitral bioprosthesis with RESILIA tissue. This trileaflet bioprosthesis is the same as the Carpentier-Edwards PERIMOUNT Magna Mitral Ease valve (Model 7300TFX; Edwards Lifesciences), except including RESILIA tissue leaflets.

Patients older than 18 years old with mitral valve disease requiring replacement based on a preoperative evaluation and scheduled to undergo MVR with or without coronary artery bypass graft were eligible. Concomitant tricuspid valve repair and the maze procedure were allowed; no other valve replacements were allowed as part of the procedure. Patients with acute myocardial infarction within 30 days before surgery, cerebrovascular accident within 6 months, ejection fraction less than 20%, or renal failure, or who required emergency surgery, were not eligible for inclusion in the study. The decision for antiplatelet or anti-coagulation (AC) therapy was not dictated per protocol but rather left to the physician's discretion per existing American College of Cardiology/American Heart Association guidelines for the management of patients with valvular heart disease.^3^ The institutional review board of each participating site approved the study protocol and publication of data. Institutional review board approval details for each center are listed in Table E1. Written informed consent, in accordance with applicable international standards and trial center regulations, was obtained from each study participant, before enrollment and any trial procedures. The patient(s) provided informed written consent for the publication of the study data.

End points were defined in consultation with the US Food and Drug Administration. An independent Clinical Events Committee adjudicated end point–related adverse events, using definitions from the established guidelines at the time of study development.^14^ Safety end points included all-cause mortality, reoperation, valve explant, thromboembolism, valve thrombosis, endocarditis, all bleeding, major paravalvular leak, hemolysis, SVD, and nonstructural valve dysfunction (NSVD). As detailed by Akins and colleagues,^14^ SVD was defined as dysfunction or deterioration involving the operated valve (exclusive of infection or thrombosis), as determined by reoperation, autopsy, or clinical investigation. Conversely, NSVD was defined as any abnormality not intrinsic to the valve itself that results in stenosis or regurgitation of the operated valve or hemolysis.^14^ Hemodynamic performance was evaluated by an independent echocardiography core laboratory (BioTelemetry Research). Hemodynamic assessments for this analysis included peak mitral valve velocity, peak and mean mitral pressure gradients, effective orifice area (EOA), Doppler velocity index (DVI), and severity of valve regurgitation. Paravalvular or transvalvular regurgitation were graded as none, trace, mild, moderate, and severe. Statistical analysis was performed by the study sponsor, 10.13039/100006520Edwards Lifesciences, per the protocol and statistical analysis plan. Descriptive summary statistics for categorical variables are the percentage of subjects with a recorded value for variables of interest. Median (interquartile range) are presented for continuous measures after assessing for normality. Kaplan–Meier analyses were undertaken on safety end points of all patients successfully implanted with the study valve. SAS, version 9.3 (SAS Institute Inc) was used for all statistical analyses. Midterm results through a data extraction date of July 21, 2022, are reported herein.

## Results

**Figure 1:**
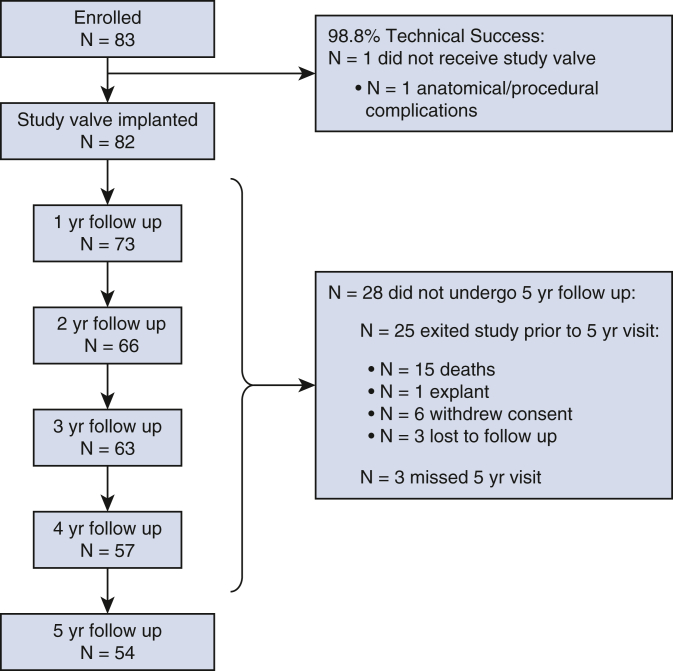
CONSORT diagram.

Between January 2013 and February 2016, 83 patients were enrolled at 17 centers in North America, with 82 patients successfully implanted with the study valve. One patient had major paravalvular leak (4+) after the heart was restarted before leaving the operating room and was deemed a technical failure; this patient was reintervened with a 27-mm Magna Mitral Ease valve. The median follow-up for the study cohort was 5.1 (1.4) years (total 374.2 patient-years in aggregate). There were 15 deaths (2 valve-related), 1 explant (due to NSVD), 1 reintervention (due to SVD), 6 who withdrew consent, 3 who were exited due to being lost to follow-up, and 3 who missed the 5-year visit, leaving 54 patients with available data at 5-year follow up (Figure 1).

Patients were predominantly elderly and had concomitant cardiovascular conditions. Median patient age at the time of the procedure was 70 years, with 52.4% of patients 70 year old and older (Table 1). The median Society of Thoracic Surgeons predicted risk of operative mortality was 3.4% (2.7%). Of the 82 patients implanted with the trial valve, the following diagnoses were present in the cohort: 48.8% pure insufficiency (n = 40/82), 30.5% stenosis with insufficiency (n = 25/82), 11.0% stenosis (n = 9/82), 7.3% prosthetic valve dysfunction (n = 6/82), and 2.4% other diagnosis (n = 2/82). A majority of patients were taking antiplatelet therapy (51.2%), and 46.3% of patients were taking anticoagulant therapy at baseline. Fewer than half of the patients (42.7%) underwent an isolated MVR. A similar number underwent MVR with concomitant procedures, including atrial ablation or left atrial appendage ligation. Of note, nearly 15% of the cohort underwent coronary artery bypass graft along with the valve procedure.

**Table 1:** Baseline patient and procedure characteristics

Variable	Median (IQR) or % patients
Number of patients successfully implanted	82
Age, y	70 (13)
STS risk score (%)	3.4 (2.7)
Sex	
Female	58.5%
Male	41.5%
Age, y	
Age 80+	14.6%
Age 70-79	37.8%
Age 60-69	26.8%
Age 50-59	18.3%
Age <50	2.4%
NYHA class	
I	6%
II	35%
III	42%
IV	17%
Concomitant procedures∗	
Coronary artery bypass grafting	14.6%
Other†	42.7%
None (isolated MVR)	42.7%
Valve size, mm	
25	7.3%
27	35.4%
29	30.5%
31	19.5%
33	7.3%

*IQR*, Interquartile range; *STS*, Society of Thoracic Surgeons; *NYHA*, New York Heart Association; *MVR*, mitral valve replacement.

∗ Patients may have undergone more than one concomitant procedure; therefore, percentages may sum to more than 100%.

† Other procedures included atrial ablation, left atrial appendage closure, maze procedure, and tricuspid valve repair.

Adverse events in the postoperative period are summarized in Table 2. All-cause mortality within 30 days of MVR was 1.2%. There were 2 early ischemic strokes related to the procedure (2.4%) and 1 early bleeding event (1.2%) due to an esophageal tear, also deemed procedure-related. No study valve explants, valve thrombosis, endocarditis, hemolysis, or reoperations were observed in the 30 days following the procedure. In addition, 68% of patients were on AC therapy at 3 months postoperatively, whereas 57.4% of patients were on AC therapy at 5 years.

**Table 2:** Safety events

Event	Early (≤30 d) N (%)	Cumulative at 5 y N	Probability event-free at 5 y % (95% CI)
All-cause mortality	1 (1.2%)	15	79.9% (70.8%-89.1%)
Reoperation	0 (0%)	2	97.1% (93.1%-100%)
Thromboembolism	2 (2.4%)	9	87.0% (78.9%-95.0%)
All bleeding	1 (1.2%)	18	74.6% (64.4%-84.9%)
Endocarditis	0 (0%)	2	96.9% (92.7%-100%)
Hemolysis	0 (0%)	0	100% (100%-100%)
Valve dysfunction			
SVD	0 (0%)	1	98.7% (96.1%-100%)
NSVD	0 (0%)	2	97.0% (92.8%-100%)
Major PVL∗	0 (0%)	0	100% (100%-100%)
Study valve explant	0 (0%)	1	98.6% (95.8%-100%)
Valve thrombosis	0 (0%)	1	98.5% (95.5%-100%)

All events as defined by Akins and colleagues^14^ and as adjudicated by Clinical Events Committee. All percentages computed as % of the total number of successfully implanted patients (N = 82). One patient was omitted from further analysis due to major PVL (4+) after the heart was restarted before leaving the operating room and was deemed a technical failure; this patient was reintervened with a 27-mm Magna Mitral Ease valve. *CI*, Confidence interval; *SVD*, structural valve deterioration; *NSVD*, nonstructural valve dysfunction; *PVL*, paravalvular leak.

∗ Major PVL = paravalvular leak of any grade requiring surgical intervention or considered a serious adverse event.

**Figure 2:**
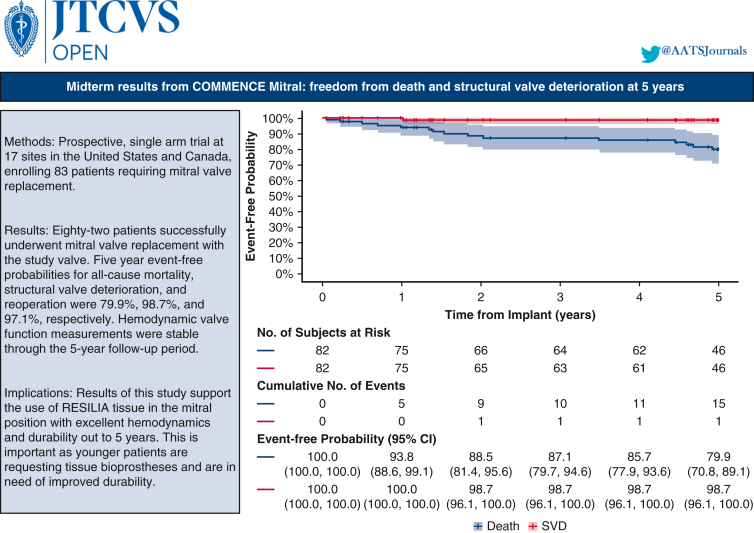
Kaplan–Meier: overall survival and freedom from SVD. *SVD*, Structural valve deterioration; *CI*, confidence interval.

Five-year event-free probabilities for all-cause mortality, SVD, and reoperation were 79.9%, 98.7%, and 97.1%, respectively (Table 2). The risk of death exceeded that of SVD throughout the follow-up period, as shown in the Kaplan–Meier curve (Figure 2). Two patients underwent reoperations: 1 for NSVD and 1 for SVD. Approximately 1-year postimplant, the patient with NSVD presented with severe mitral regurgitation. The posterior leaflet was thickened and stuck in intermediate position resulting in a severe, eccentric leak. The patient was reintervened with a 29-mm Magna Mitral Ease valve (Edwards Lifesciences) shortly thereafter. Upon postexplant investigation, the valve had signs of pannus overgrowth on both leaflets with no evidence of SVD. The observed SVD occurred in a 77 year-old patient implanted with the trial valve. The patient presented with end-stage renal disease on hemodialysis and underwent valve-in-valve with a 29-mm SAPIEN 3 (Edwards Lifesciences) transcatheter heart valve on postoperative day 638 for severe central regurgitation. The second NSVD was observed in a patient who was hospitalized for chronic heart failure in the setting of severe right ventricular dysfunction. Clinically, the patient presented with mild-to-moderate stenosis seen functionally as elevated mitral valve pressure gradients, which the clinical events committee deemed as NSVD due to study valve stenosis. The patient did not require reintervention as of the 5-year follow-up visit.

**Figure 3:**
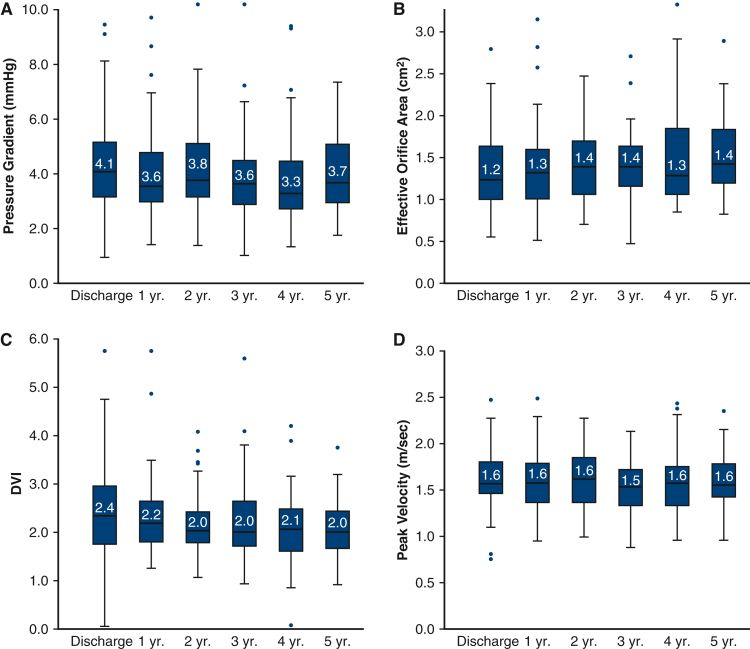
Echocardiography-derived valve hemodynamic outcomes over the follow-up period: A, Mean mitral pressure gradients; B, effective orifice area; C, Doppler velocity index (*DVI*); and D, peak velocity.

**Figure 4:**
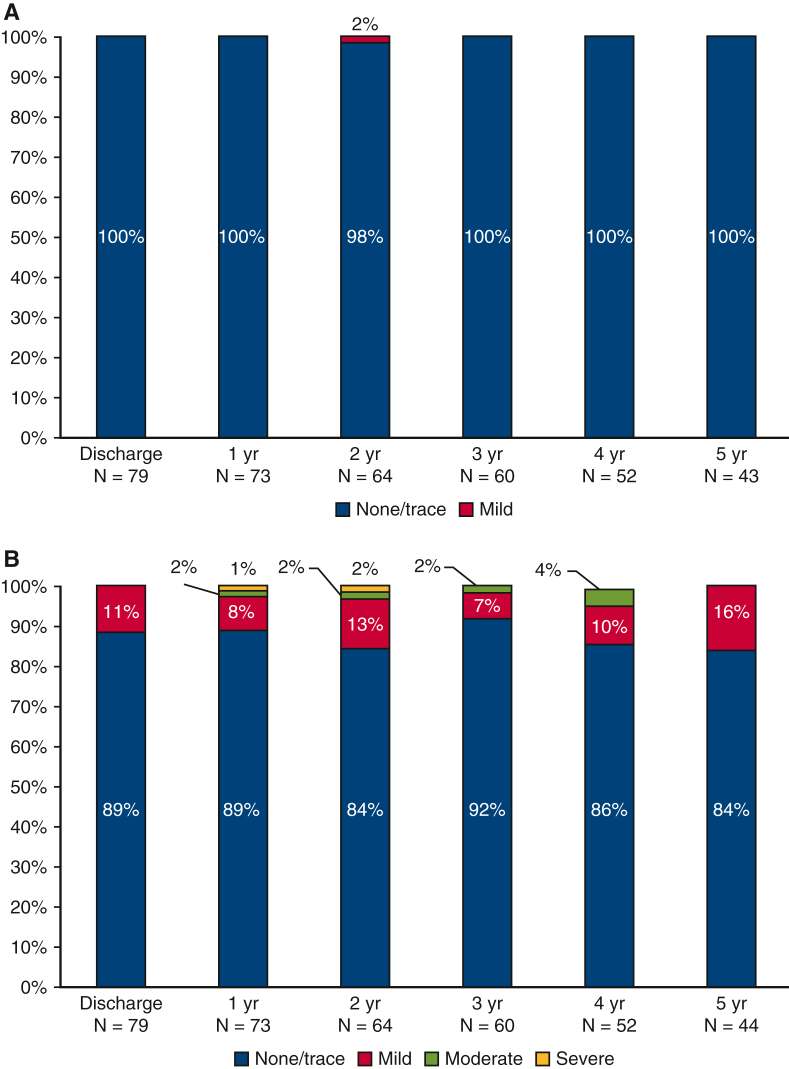
Paravalvular (A) and transvalvular (B) leak during the study period.

A variety of valve function measurements were evaluated by echocardiography (Figure 3). Stable pressure gradients were observed from discharge to 5 years (4.1 [2.0] and 3.7 [2.2] mm Hg, respectively). Peak mitral velocity was also stable during the following up period (1.6 [0.4] m/s, discharge; 1.6 [4.0] m/s, 5 years). Effective orifice areas (EOAs) were lower than expected during the follow-up period (1.2 [0.6] cm^2^, discharge; 1.4 [0.6] cm^2^, 5 years). Given that literature suggests the continuity equation may underestimate EOAs due to variability in left ventricular outflow tract diameter measurements, DVI is presented to supplement the EOA data.^15^^,^^16^ From discharge to 5 years follow-up, DVI was stable (2.4 [1.3] and 2.0 [0.8], respectively) and largely within expected range. New York Heart Association class improved universally after MVR, and 94% of patients were maintained in New York Heart Association class I or II through 5 years of follow-up. In addition, clinically insignificant trivial to mild paravalvular or transvalvular leak was predominantly observed. (Figure 4).

## Discussion

Through 5 years of follow-up, the COMMENCE Mitral trial reported a good safety profile and clinically stable hemodynamic performance of a bioprosthetic valve with RESILIA tissue. These findings are consistent with the midterm safety and efficacy outcomes observed with the RESILIA tissue in the aortic position. Durability results are promising with 2 observed reoperations (1 NSVD, 1 SVD) and a reported freedom from SVD at 5 years of 98.7%. Mitral valve pressure gradients are the most common measure of valve function, whereas EOAs are more difficult to measure and may be less predictive of valve dysfunction.^17^ As such, DVI was also included as an additional measure to document any significant change or deterioration in valve function. Hemodynamics were clinically stable, as measured by mean valve gradients, peak velocities, DVI, and EOAs.

In surgical literature, several studies have reported mid-term MVR outcomes for bioprosthetic valves.^18^, ^19^, ^20^, ^21^ A comparative analysis of 940 MVR patients implanted with either Medtronic Mosaic or PERIMOUNT mitral valves reported 5-year rates of echocardiographic freedom from SVD of 91.0% and 90.3%, respectively.^18^ Further, publications for the PERIMOUNT Magna, Medtronic Mosaic, and Abbott Epic bioprosthetic valves reported 5-year rates of freedom from SVD of 90%, 94%, and 93% respectively.^19^, ^20^, ^21^ It is important to note that all of these studies represent single-center retrospective studies without clinical events committee adjudicated safety end points or core laboratory–adjudicated echo data. In addition, study cohort characteristics need to be carefully considered when comparing these results (eg, demographics, study location). Lastly, the interpretation of the Akins definition varies greatly from study to study.

There have been very few clinical trials in the MVR space over the last 25 years.^22^, ^23^, ^24^ As such, COMMENCE Mitral is unique in that it reports the first clinical data of RESILIA tissue in the mitral position and is a prospectively collected MVR clinical trial. Given the level of scientific rigor in this trial, the observed 98.7% freedom from SVD rate compares favorably relative to other published mid-term MVR studies. The patients in COMMENCE Mitral are demographically similar when compared with MVR populations in published literature.^8^^,^^18^ Surgical outcomes were excellent compared with risk scores, allowing more focus on actual valve function. The trial data set followed more standardized definitions for safety events than a retrospective single-center analysis and were adjudicated by a clinical events committee. In addition, all echocardiography results were adjudicated rigorously by an independent echo-core laboratory, and high compliance with follow-up visits was achieved (95%). Overall, the study is encouraging for durability of RESILIA tissue in the mitral position.

Although this trial presents highly relevant outcomes, it is not without limitations. This study is limited by small cohort size. Further, the study reports midterm MVR outcomes, which is notable, since several studies show a significant increase in valve deterioration beginning after 5 years.^18^, ^19^, ^20^, ^21^, ^22^^,^^25^ As a result, long-term follow-up is needed to understand whether these findings are maintained beyond 5 years. This limitation will be addressed in part when outcomes from the 10-year COMMENCE trial extended follow-up cohort are evaluated. Further, real-world data on acute and long-term safety and performance of the MITRIS RESILIA Mitral valve will be collected in the prospective, global MOMENTIS trial with outcomes reported through 10 years' postimplant (https://clinicaltrials.gov/ct2/show/NCT05526560).

The clinical implications of this study support the use of RESILIA tissue in the mitral position with excellent hemodynamics and durability out to 5 years. This is important as younger patients are requesting tissue bioprostheses and are in need of improved durability. The MITRIS RESILIA mitral valve is now available for clinical use in the United States and Japan and will contribute to further information on durability.

## Conclusions

Through 5 years of follow-up, the COMMENCE Mitral trial reported a good safety profile and clinically stable hemodynamic performance of a bioprosthetic valve with RESILIA tissue. Longer-term follow up is needed to fully assess durability of this novel option for mitral valve replacement.

### Webcast

You can watch a Webcast of this AATS meeting presentation by going to: https://www.dropbox.com/s/0u5f52sxsvyrv06/__Full%20vid%20for%20Sara%20-%20AM21_A20%20-%20Innovations%20in%20Valve%20Technology%20and%20Pros%20-%20FULL.mp4?dl=0.

**Figure:**
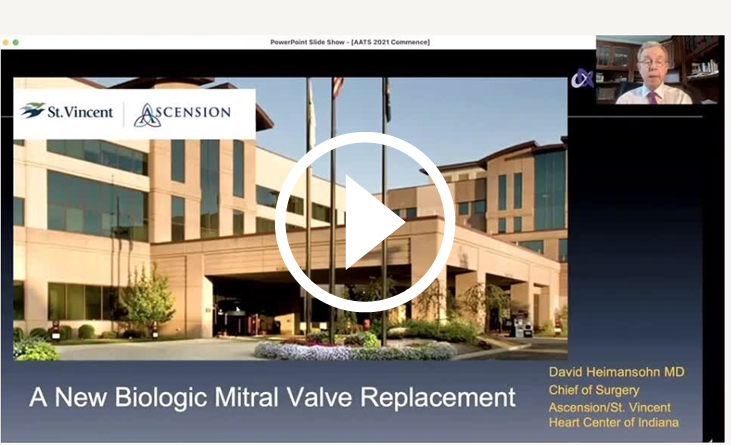

### Conflict of Interest Statement

E.R. reported research/consulting fees from Abbott, Edwards Lifesciences, Boston Scientific, and AtriCure. H.T. reported consultant, Edwards Lifesciences. F.D. reported consultant, Cook Medical; and honoraria, Edwards Lifesciences and Medtronic. M.A.M. reported consultant, honoraria, and research with Medtronic, Edwards Lifesciences, ZMedica, AtriCure, JOMDD, and Abbott Laboratories. P.P. reported funding from 10.13039/100006520Edwards Lifesciences, 10.13039/100004374Medtronic, Pi-Cardia, and Cardiac Success for echocardiography core laboratory analyses and research studies in the field of transcatheter valve therapies, for which he received no personal compensation; and lecture fees from Edwards Lifesciences and Medtronic. All other authors reported no conflicts of interest.

The *Journal* policy requires editors and reviewers to disclose conflicts of interest and to decline handling or reviewing manuscripts for which they may have a conflict of interest. The editors and reviewers of this article have no conflicts of interest.

## Figures and Tables

**Table E1:** Institutional review board approvals

Site #	Site name Address	Principal Investigator	IRB/EC name Address	IRB/EC approval date	IRB approval number
083	AdventHealth Orlando 601 E. Rollins St, MB #99 Orlando, FL 32803	Kevin Accola, MD	AdventHealth Institutional Review Board Orlando 901 N. Lake Destiny Road, Suite 400 Maitland, FL 32751	24-Jan-14	Ref. # 0530, Proj#: 507,204-4
169	Mount Sinai Hospital Gustave L. Levy Place ICAHN School of Medicine 1190 Fifth Avenue, 2nd Floor New York, NY 10029	David Adams, MD	Icahn School of Medicine at Mount Sinai One Gustave L. Levy Place, Box 1081 New York, NY 10029	27-Aug-13	Proj#: HS#: 13-00479 GCO#:13-1233(0001) (01)
014	Institut Universitaire de Cardiologie et de Pneumologie De Québec (IUCPQ) 2725 Chemin Ste-Foy Québec (Québec) G1V4G5	François Dagenais, MD	Centre De Recherche Institut Universitaire de Cardiologie et de Pneumologie de Québec 2725 Chemin Ste-Foy Québec (Québec) G1V4G5	22-Sep-14 *Health Canada Approval* *26-May-15*	Ref Number: 2015-2394, 21,104—3000
180	University of Maryland School of Medicine 110 S. Paca St, 7th Floor Baltimore, MD 21201	Bartley Griffith, MD Previous PI: James S. Gammie, MD	University of Maryland, Baltimore Institutional Review Board 620 W. Lexington St 2nd Floor Baltimore, MD 21201	14-Mar-13	Proj#: HP-00054452
479	New York Presbyterian Weill Cornell Medical College 525 East 68th St New York, NY 10065	Leonard Girardi, MD	Weill Cornell Medicine Institutional Review Board 575 Lexington Avenue 9th Floor, New York, NY 10022	21-May-13	Protocol #: HP-00054452
085	St. Vincent’s Hospital 10590 North Meridian St. Suite 105 Indianapolis, IN 46290	David Heimansohn, MD	St. Vincent Hospital IRB Research & Regulatory Affairs 8402 Harcourt Rd, Suite 201 Indianapolis, IN 46260	14-Nov-12	Protocol#: R2012-146
718	Princeton Baptist Med Ctr 701 Princeton Ave SW Birmingham, AL 35211 Research Office Location: Cardiology P.C. 801 Princeton Avenue SW, Suite 707 Birmingham, AL 35211	Thomas Cawthon Jr, MD Previous PI: Clifton Lewis, MD	Metro West Medical Center IRB 115 Lincoln St Framingham, MA 01702-9167	13-Mar-15	Proj#: S-1068
717	Ohio Health Research Institute 3545 Olentangy River Rd Columbus, OH 43214	Jeffrey Lyons, MD	WIRB-Copernicus Group Institutional Review Board (WCG IRB) 1019 39th Ave SE Suite 120 Puyallup, WA 98374-2115	16-Apr-15	Proj# 1154021, 20,130,138
394	Yale University 47 College St, Suite 203 New Haven, CT 06510-3209 Research Office Location: 330 Cedar St, Boardman Building, Suite #204 New Haven, CT 06510	Abeel Mangi, MD *Previous PI: Sabet Hashim, MD*	Human Investigation Committee 25 Science Park—3rd Fl., 150 Munson St New Haven, CT 06520-8327	17-Dec-14	Protocol# 1406014165
176	Washington University Barnes-Jewish Hospital Southwest Tower, POD 3 1 Barnes-Jewish Hospital Plaza St. Louis, MO 63110	Spencer Melby, MD	Washington University Human Research Protection Office 660 South Euclid Ave Campus Box 8089 St. Louis, MO 63110	22-Oct-15	ID# 201509142
407	Pinnacle Health at Harrisburg Hospital 111 South Front St Harrisburg, PA 17101 Research Office Location: Pinnacle Health Cardiovascular Institute 1000 North Front St Wormleysburg, PA 17043	Mubashir Mumtaz, MD	UPMC Pinnacle IRB 307 South Front St Press Hall, First Floor Harrisburg, PA 17104	11-Feb-14	PHH#: 13-065
699	Mount Sinai Beth Israel Beth Israel Medical Center 10 Nathan D. Perlman Pl. New York, NY 10003 Research Office Location: 350 East 17th St, 5th FL New York, NY 10003	John Puskas, MD	Icahn School of Medicine at Mount Sinai One Gustave L. Levy Place, Box 1081 New York, NY 10029	28-Oct-14	HS#: 13-00479, GCO#1: 13-1233(0001)
367	St. Thomas Health 4220 Harding Rd Nashville, TN 37205 Research Office Location: 4230 Harding Road, Ste 430 Nashville, TN 37205	Evelio Rodriguez, MD	Sterling IRB 6300 Powers Ferry Rd Suite 600-351 Atlanta, GA 30,339	29-Jan-15	ID#: 4976
532	University of Southern California—Department of Cardiothoracic Surgery 1520 San Pablo St, Ste 4300 Los Angeles, CA 90033	Craig Baker, MD Previous PI: Vaughn Starnes, MD PhD	University of Southern California Health Sciences Campus Institutional Review Board LAC + USC Medical Center, General Hospital Suite 4700 1200 North State St, Los Angeles, CA 90033	04-Mar-13	Proj# HS-13-00,059
183	New York Presbyterian Hospital—Columbia University Medical Center 177 Fort Washington Ave, Suite 7-435 New York, NY 10032	Hiroo Takayama, MD PhD *Previous PI: Alan Stewart MD*	Columbia University Institutional Review Board 154 Haven Ave, 1 Floor New York, NY 10032	19-Nov-12	Protocol# IRB-AAAK8159
715	North Mississippi Medical Center, Inc. 830 South Gloster St Fourth Floor East Tower Tupelo, MS 38801 Research Office Address: Cardiology Associates Research LLC 830 South Gloster St Third Floor East Tower Tupelo, MS 38801	David Talton, MD	North Mississippi Health Services IRB 830 South Gloster St Tupelo, MS 38801	20-Jan-15	Not Applicable
681	Cardiac Surgery Clinical Research Center, Inc (Advocate Christ) 4400 W. 95th St, Ste 205 Oak Lawn, IL 60453	Antone Tatooles, MD	Advarra 1501 Fourth Ave, Suite 800 Seattle, WA 98101	10-Oct-14	Proj#AHC-5803, File#: 29,327/1

*IRB/EC*, Institutional review board/ethics committee.
